# Supplementary figures and images for: AutoPhy: Automated phylogenetic identification of novel protein subfamilies
Source: PLoS One. 2024 Jan 11;19(1):e0291801. doi: 10.1371/journal.pone.0291801 (PMC10783759; doi:10.1371/journal.pone.0291801)

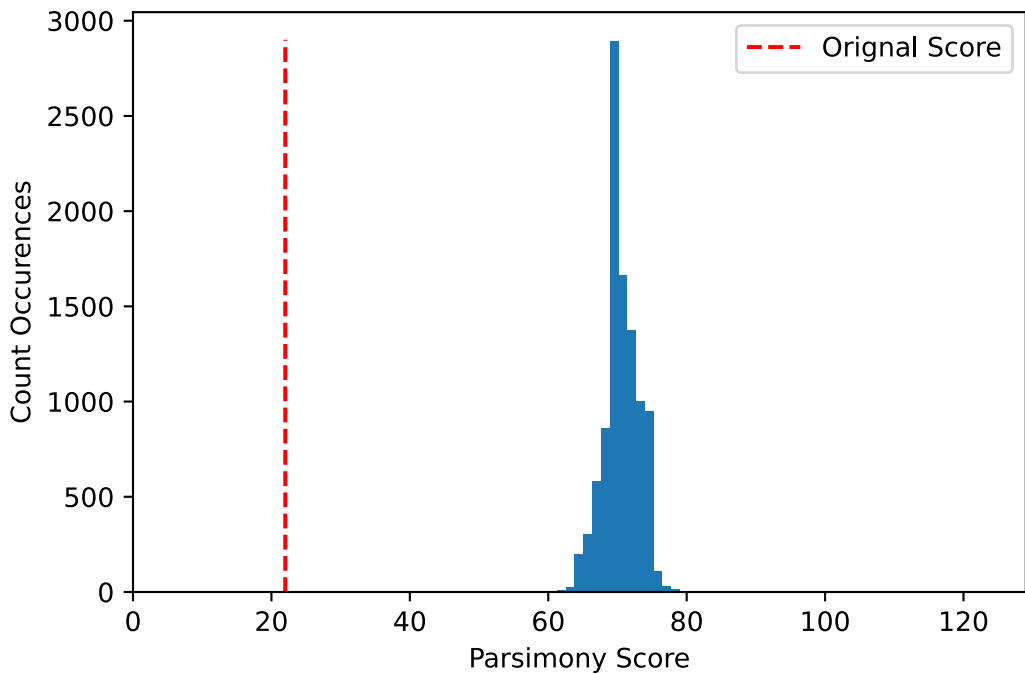

Supplement: S6 Fig — Dashed red indicates the original tree–s score. (PDF) [file pone.0291801.s006.pdf]

A

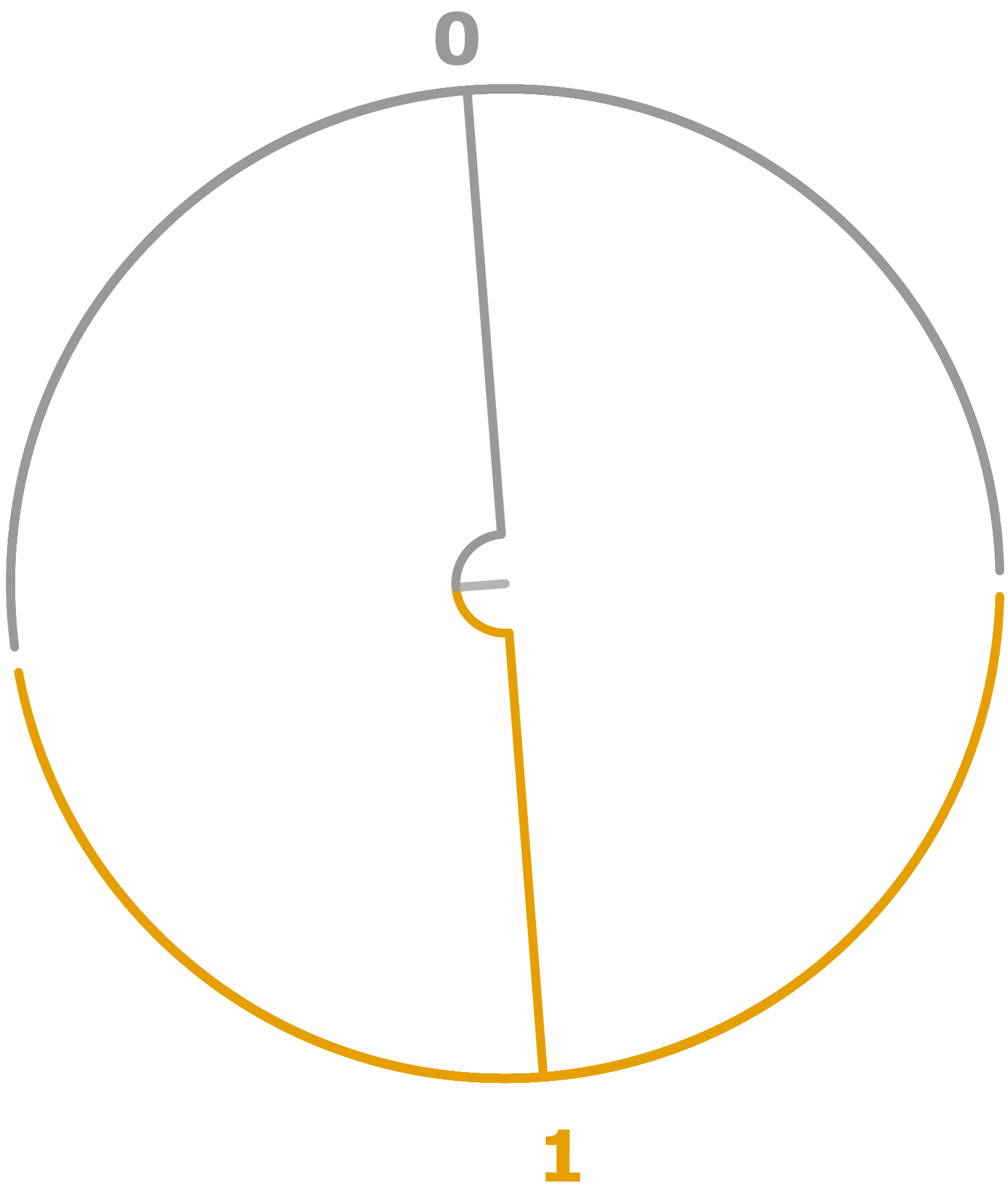

B

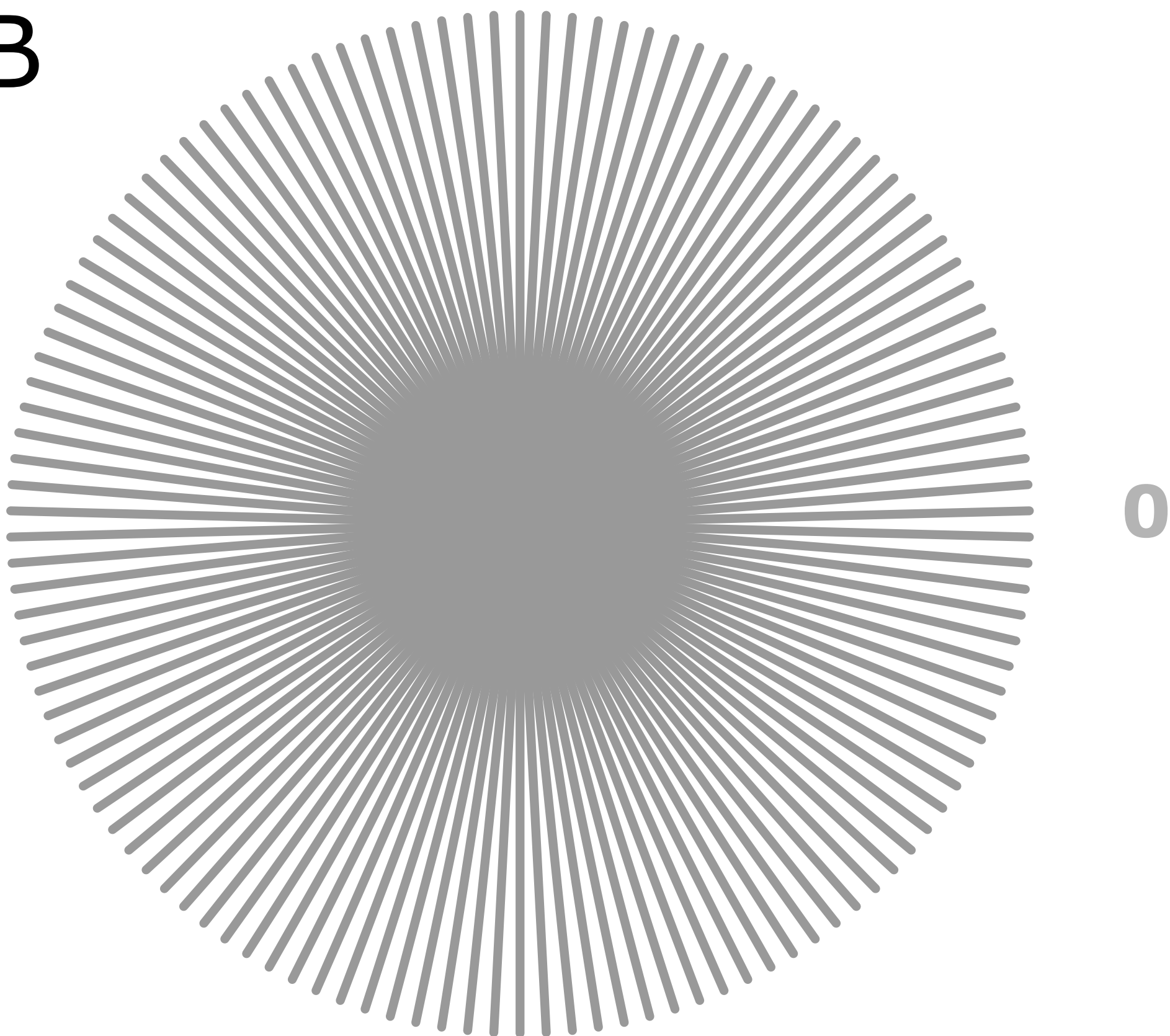

Supplement: S7 Fig — (PDF) [file pone.0291801.s007.pdf]
